# Temporal Trends in Antimicrobial Resistance and Risk Factors in Patients with Pyogenic Liver Abscess: A Four-Year Cross-Sectional Study: Resistance Trends in Pyogenic Liver Abscess

**DOI:** 10.31661/gmj.vi.3873

**Published:** 2025-06-29

**Authors:** Jie Liu, Fengmei Wang

**Affiliations:** ^1^ The Third Central Clinical College of Tianjin Medical University, Tianjin, China; ^2^ Tianjin Key Laboratory of Extracorporeal Life Support for Critical Diseases, Artificial Cell Engineering Technology Research Center, Tianjin Institute of Hepatobiliary Disease, Tianjin, China; ^3^ Tianjin Key Laboratory of Molecular Diagnosis and Treatment of Liver Cancer, Tianjin First Center Hospital, Tianjin, China

**Keywords:** Pyogenic Liver Abscess, Klebsiella Pneumoniae, Antimicrobial Resistance

## Abstract

**Background:**

Pyogenic liver abscess (PLA) is a serious liver infection, particularly common in patients with immune deficiencies or chronic liver diseases. Given the rising antimicrobial resistance in PLA-associated pathogens, this study aimed to investigate the temporal trends in antimicrobial resistance and associated risk factors in PLA patients over a four-year period.

**Materials and Methods:**

This cross-sectional study was conducted at Tianjin First Center Hospital, China, by reviewing the medical records of PLA patients from January 2019 to December 2023. Patients were categorized into two groups: Klebsiella pneumoniae (KPLA) and non-Klebsiella pneumoniae (NKPLA). Data on clinical characteristics, laboratory results, imaging findings, microbiological culture results, and antimicrobial susceptibility patterns were collected. Temporal changes in antimicrobial resistance and associated risk factors were analyzed.

**Results:**

A total of 328 patients were included, of whom 297 (90.5%) were infected with Klebsiella pneumoniae (KPLA) and 31 (9.5%) with non-Klebsiella pathogens (NKPLA). Over the four-year period, the number of PLA cases gradually increased, with K. pneumoniae remaining the predominant pathogen. Resistance to ceftriaxone (20% in 2019 and 20% in 2023) and ciprofloxacin (25% in 2019 and 23% in 2023) showed slight variations, while carbapenem resistance decreased (imipenem: 7% in 2019 and 4% in 2023, meropenem: 6% in 2019 and 4% in 2023). In comparing the KPLA and NKPLA groups, KPLA patients were more likely to have diabetes (61.6% vs. 38.7%, P=0.01) and cryptogenic origin (65.7% vs. 41.9%, P0.05). NKPLA cases had higher prevalence of hepatobiliary disease (32.3% vs. 18.2%, P=0.04) and malignancy (19.4% vs. 8.8%, P=0.03).

**Conclusion:**

This study highlights the increasing antimicrobial resistance in K. pneumoniae and E. coli associated with PLA, underscoring the need for updated local antibiograms and revised empirical therapy protocols. Furthermore, the clinical and microbiological differences between KPLA and NKPLA suggest that pathogen-specific and resistance-based stratification should replace traditional classifications.

## Introduction

Pyogenic liver abscess (PLA) is a serious infectious disease of the liver that can lead to significant inflammation and tissue destruction, potentially resulting in severe complications or even death [1].

Multiple bacterial pathogens, including Klebsiella pneumoniae and Escherichia coli, are commonly implicated in the development of PLA [2][3]. This condition is particularly prevalent in patients with immune deficiencies or chronic liver diseases, especially in developing countries [4]. Furthermore, the increasing prevalence of antimicrobial resistance in PLA-associated pathogens poses considerable therapeutic challenges, highlighting the need for more detailed epidemiological investigations [5][6].

Previous studies have shown a rising trend in antibiotic resistance in Klebsiella pneumoniae, a major causative agent of PLA, with this trend continuously changing in certain regions [7][8].

However, most existing studies have focused on cross-sectional data obtained at a specific point in time, with limited attention paid to temporal changes in antimicrobial resistance and the associated risk factors over time [9].

The aim of this study is to analyze the temporal changes in antimicrobial resistance in the pathogens responsible for PLA over a four-year period. This research will be conducted using a cross-sectional study design, with data collected from patients admitted to hospitals during this period.

Specifically, the study will focus on examining changes in resistance to various antibiotics, with an emphasis on factors such as pathogen type, clinical characteristics of patients, and environmental risk factors. Additionally, epidemiological evidence will be analyzed to assess temporal trends in antimicrobial resistance and to identify emerging resistance patterns, particularly in Klebsiella pneumoniae and Escherichia coli [10][11].

This study is expected to provide valuable insights into the evolving patterns of antimicrobial resistance in PLA and the associated risk factors, offering potential strategies for improving treatment and prevention of this serious liver infection in the future.

## Materials and Methods

This retrospective cross-sectional study was conducted at Tianjin First Center Hospital, a tertiary care teaching hospital in China, between January 2019 and December 2023. Patients diagnosed with PLA were enrolled through review of the hospital’s electronic medical records system. The study protocol was approved by the Ethics Committee of Tianjin University (Approval No. BER-YXY-2024020).

The inclusion criteria consisted of patients diagnosed with pyogenic liver abscess between January 2019 and December 2023 based on clinical manifestations such as fever, chills, and right upper quadrant pain supported by laboratory data and imaging findings from ultrasound or CT scans, along with available microbiological culture results from pus or blood samples and complete clinical records including demographic information, comorbidities, laboratory values, treatment details, and outcomes, while exclusion criteria involved non-bacterial liver abscesses caused by fungi, parasites or Mycobacterium tuberculosis as well as patients with incomplete medical records that prevented proper evaluation.

For each patient, data were extracted regarding age, sex, comorbidities (e.g., diabetes, hypertension), clinical symptoms, laboratory values, imaging findings, microbiological culture results (blood and/or abscess drainage), antibiotic susceptibility patterns, treatment modalities, and clinical outcomes.

All bacterial isolates were identified using automated systems, primarily the VITEK-2 Compact system (bioMérieux, France) with version 9.01 software.

Antimicrobial susceptibility testing was carried out according to the Clinical and Laboratory Standards Institute (CLSI) guidelines, M100, 30th edition (2020). Quality control strains such as Escherichia coli ATCC 25922 and Staphylococcus aureus ATCC 29213 were used to ensure the accuracy of the results. Resistance profiles were confirmed by manual methods such as disk diffusion or E-test when necessary.

Based on microbiological culture results, and given that Klebsiella pneumoniae was the predominant pathogen among PLA cases, patients were categorized into two groups according to the presence or absence of K. pneumoniae:

- Klebsiella pneumoniae liver abscess (KPLA): patients with K. pneumoniae isolated, either as a single organism or part of polymicrobial infection

- Non-Klebsiella PLA (NKPLA): patients in whom K. pneumoniae was not isolated

Temporal trends in antibiotic resistance were analyzed over the five-year period. In the NKPLA group, due to the limited number of cases with each specific pathogen, all non-Klebsiella isolates were grouped together for statistical comparison, although individual pathogen frequencies were also reported descriptively.

Subgroup analyses were conducted to explore associations between antibiotic resistance and clinical variables such as diabetes mellitus, prior antibiotic use, and presence of sepsis.

### Statistical Analysis

Descriptive statistics were used to summarize patient characteristics. Continuous variables were expressed as mean ± standard deviation or median (interquartile range), and compared using t-tests or Mann-Whitney U tests depending on distribution. Categorical variables were presented as counts and percentages and compared using Chi-square tests or Fisher’s exact tests as appropriate. To evaluate trends in antibiotic resistance over time, Chi-square test for trend and Cochran-Armitage trend test were used.

Furthermore, multivariate logistic regression was employed to identify independent risk factors associated with antimicrobial resistance, adjusting for confounders such as age, comorbidities, and clinical presentations. A P-value < 0.05 was considered statistically significant. All analyses were performed using SPSS version 26.0 (IBM Corp., Armonk, NY, USA).

## Results

A total of 328 patients with pyogenic liver abscess (PLA) were included in this study. Among them, 297 (90.5%) were infected with Klebsiella pneumoniae (KPLA group), and 31 (9.5%) were infected with non-Klebsiella pathogens (NKPLA group).

### Temporal Trends

Over the 4-year study period (2019-2023), the number of PLA cases showed a gradual increase, with K. pneumoniae remaining the predominant pathogen each year. The proportion of KPLA increased from 87.0% in 2019 to 93.4% in 2023 (Table-1).

### Antimicrobial Resistance Trends

The overall resistance of K. pneumoniae to commonly used antibiotics was assessed over the five-year period. Resistance rates to third-generation cephalosporins (ceftriaxone and ceftazidime) remained relatively stable, while a gradual decrease in resistance to carbapenems (imipenem and meropenem) was observed in recent years. Although resistance percentages are presented in the table, the actual number of resistant isolates per year was small and thus rounded percentages were used to avoid fractional sample sizes (Table-2).

### Risk Factor Comparison

Patients with KPLA were more likely to have diabetes mellitus (62% vs. 39%, P=0.01) and cryptogenic origin (66% vs. 42%, P<0.05) than NKPLA patients. NKPLA cases showed a higher prevalence of hepatobiliary disease and malignancy (Table-3). (All percentages were rounded to the nearest whole number to reflect actual patient counts.)

## Discussion

**Table:** Table1. Annual Distribution of PLA Cases and K. pneumoniae Proportions (2019-2023)

**Year**	**Total PLA cases**	**KPLA cases**	**NKPLA cases**	**Proportion of KPLA (%)**
**2019**	77	67	10	87
**2020**	66	60	6	90.9
**2021**	62	56	6	90.3
**2022**	58	53	5	91.4
**2023**	65	61	4	93.8

**Table:** Table2. Antimicrobial Resistance Rates of K. pneumoniae Over Time (2019-2023)

**Antibiotic**	**2019**	**2020**	**2021**	**2022**	**2023**
					
**Ceftriaxone**	18%	20%	21%	19%	20%
**Ceftazidime**	16%	17%	18%	17%	16%
**Imipenem**	7%	6%	6%	5%	4%
**Meropenem**	6%	5%	5%	4%	4%
**Ciprofloxacin**	25%	26%	27%	24%	23%

**Table:** Table3. Comparison of Clinical Characteristics between KPLA and NKPLA Patients

**Variable**	**KPLA (n=297)**	**NKPLA (n=31)**	**P-value**
**Age (years, mean ± SD)**	59.3 ± 12.4	61.7 ± 11.9	0.29
**Male gender (%)**	68%	65%	0.68
**Diabetes mellitus (%)**	62%	39%	0.01
**Biliary disease (%)**	18%	32%	0.04
**Malignancy (%)**	9%	19%	0.03
**Cryptogenic PLA (%)**	66%	42%	0.02

Liver abscess (PLA) continues to be a significant clinical issue, carrying a potential risk to life, despite the advancements in diagnostic techniques and antimicrobial treatments. Our cross-sectional study, conducted between 2019 and 2023, reveals shifting trends in antimicrobial resistance (AMR) among the main pathogens responsible for PLA, particularly focusing on Klebsiella pneumoniae and Escherichia coli. In the present study, Escherichia coli, which was the second most common pathogen, showed a higher resistance to a variety of antibiotics compared to Klebsiella pneumoniae. Specifically, resistance rates to β-lactam/β-lactamase inhibitor combinations and fluoroquinolones increased progressively between 2019 and 2023, mirroring the trends observed in national surveillance reports from China [18][19]. These findings emphasize the need to revisit and update empirical treatment guidelines, particularly for patients with biliary tract infections, which were more often associated with E. coli in our cohort. Moreover, the persistent escalation of E. coli resistance underscores the critical need for local antibiograms to be revised and empirical treatment protocols adjusted accordingly.

As previously reported in East Asian epidemiological studies, especially in China, Klebsiella pneumoniae remained the leading pathogen throughout the study period [12][13][14][15]. The predominance of K. pneumoniae in PLA cases is largely attributed to its colonization of the gastrointestinal tract and its ability to cause severe invasive infections, particularly in patients who are diabetic or immunocompromised [16]. Although we observed a consistent dominance of K. pneumoniae, subtle shifts in resistance patterns were noted, with a statistically significant rise in resistance to third-generation cephalosporins (such as cefotaxime and ceftazidime) and fluoroquinolones over time (P<0.05), which raises concerns about the adequacy of empirical therapies [17].

An important observation was the generally stable resistance of both K. pneumoniae and E. coli to carbapenems. However, the emergence of carbapenem-resistant K. pneumoniae strains was seen after 2021, aligning with other recent reports from China that suggest a gradual, but worrying, increase in carbapenem resistance in community-acquired PLA [20][21].

Given the high virulence and potential for severe infections caused by carbapenem-resistant strains, it is critical to ensure stringent antibiotic stewardship and consistent adjustments to therapy based on culture results.

To provide a broader context for our study's findings, we compared them with prior large-scale studies. For instance, a study by Siu et al. (PMC5144064) recorded 296 cases of Klebsiella pneumoniae liver abscess over a period of more than 10 years. In contrast, our study identified 297 cases of KP-PLA in just five years, reflecting a notable increase in case volume.

This discrepancy may be attributed to regional epidemiological variations, better awareness of the disease, improvements in diagnostic techniques (such as advanced imaging and automated culture systems), and enhanced detection due to electronic health records. The higher prevalence of diabetes and the aging population in our area may also contribute to the observed rise in cases. These factors highlight the importance of utilizing local data to inform treatment and prevention strategies [22].

From a demographic standpoint, the clinical characteristics of PLA patients remained largely consistent throughout the study period, with a predominance of males and most patients being over 60 years of age. The right hepatic lobe was the most common site for abscess formation, which may be attributed to both anatomical and hemodynamic factors [23].

In conclusion, the progressive increase in antimicrobial resistance among PLA pathogens, particularly E. coli, emphasizes the urgent need for regular updates to local antibiograms and tailored empirical treatment strategies. Continued surveillance and molecular investigations into resistance mechanisms should be integral components of infection control protocols within hospitals, to prevent the further spread of multidrug-resistant PLA.

## Conclusion

This cross-sectional study provides updated insights into the temporal trends of antimicrobial resistance (AMR) among pathogens causing PLA in a tertiary center over a four-year period. Klebsiella pneumoniae remained the predominant pathogen, followed by Escherichia coli.

Although K. pneumoniae exhibited relatively low resistance rates to most antibiotics, a slight upward trend in resistance to third-generation cephalosporins and fluoroquinolones was observed. E. coli demonstrated a higher initial resistance rate compared to K. pneumoniae, particularly to beta-lactam/beta-lactamase inhibitors and fluoroquinolones, with a gradual increase over the years.

The findings highlight the necessity of tailoring empirical antibiotic regimens based on local and up-to-date resistance data. Furthermore, the traditional binary classification of PLA into KPLA and NKPLA may be inadequate due to the heterogeneity of the NKPLA group. Pathogen-specific analysis should be prioritized for accurate interpretation and clinical decision-making. Continuous microbiological surveillance and rational antibiotic stewardship are essential to mitigate the rise of drug-resistant pathogens in PLA.

## Conflict of Interest

The authors declare that they have no conflict of interest

## References

[R1] Kaplan GG, Gregson DB, Laupland KB (2004). Population-based study of the epidemiology of and the risk
factors for pyogenic liver abscess. Clinical Gastroenterology and Hepatology.

[R2] Qu TT, Zhou JC, Jiang Y, Shi KR, Li B, Shen P, Wei ZQ, Yu YS (2015). Clinical and microbiological characteristics of Klebsiella
pneumoniae liver abscess in East China. BMC infectious diseases.

[R3] Serraino C, Elia C, Bracco C, Rinaldi G, Pomero F, Silvestri A, Melchio R, Fenoglio LM (2018). Characteristics and management of pyogenic liver abscess: A
European experience. Medicine.

[R4] Meddings L, Myers RP, Hubbard J, Shaheen AA, Laupland KB, Dixon E, Coffin C, Kaplan GG (2010). A population-based study of pyogenic liver abscesses in the
United States: incidence, mortality, and temporal trends. Official journal of the American College of Gastroenterology| ACG.

[R5] Fung CP, Lin YT, Lin JC, Chen TL, Yeh KM, Chang FY, Chuang HC, Wu HS, Tseng CP, Siu LK (2012). Klebsiella pneumoniae in gastrointestinal tract and pyogenic
liver abscess. Emerging infectious diseases.

[R6] Zhang S, Zhang X, Wu Q, Zheng X, Dong G, Fang R, Zhang Y, Cao J, Zhou T (2019). Clinical, microbiological, and molecular epidemiological
characteristics of Klebsiella pneumoniae-induced pyogenic liver abscess in
southeastern China. Antimicrobial Resistance & Infection Control.

[R7] Liu J, Liu Y, Li C, Peng W, Jiang C, Peng S, Fu L (2024). Characteristics of Klebsiella pneumoniae pyogenic liver abscess
from 2010–2021 in a tertiary teaching hospital of South China. Journal of Global Antimicrobial Resistance.

[R8] Siu LK, Yeh KM, Lin JC, Fung CP, Chang FY (2012). Klebsiella pneumoniae liver abscess: a new invasive syndrome. The Lancet infectious diseases.

[R9] Tian LT, Yao K, Zhang XY, Zhang ZD, Liang YJ, Yin DL, Lee L, Jiang HC, Liu LX (2012). Liver abscesses in adult patients with and without diabetes
mellitus: an analysis of the clinical characteristics, features of the
causative pathogens, outcomes and predictors of fatality: a report based on
a large population, retrospective study in China. Clinical Microbiology and Infection.

[R10] Lin YT, Wang FD, Wu PF, Fung CP (2013). Klebsiella pneumoniae liver abscess in diabetic patients:
association of glycemic control with the clinical characteristics. BMC infectious diseases.

[R11] Wang JH, Liu YC, Lee SS, Yen MY, Chen YS, Wang JH, Wann SR, Lin HH (1998). Primary liver abscess due to Klebsiella pneumoniae in Taiwan. Clinical Infectious Diseases.

[R12] Kaplan GG, Gregson DB, Laupland KB (2004). Population-based study of the epidemiology of and the risk
factors for pyogenic liver abscess. Clin Gastroenterol Hepatol.

[R13] Qu TT, Zhou JC, Jiang Y, Shi KR, Li B, Shen P, Wei ZQ, Yu YS (2015). Clinical and microbiological characteristics of Klebsiella
pneumoniae liver abscess in East China. BMC infectious diseases.

[R14] Serraino C, Elia C, Bracco C, Rinaldi G, Fiore M, Fagoonee S, et al (2018). Characteristics and management of pyogenic liver abscess: A
European experience. Medicine (Baltimore).

[R15] Meddings L, Myers RP, Hubbard J, Shaheen AA, Kaplan GG, Stelfox HT, et al (2010). A population-based study of pyogenic liver abscesses in the
United States: incidence, mortality, and temporal trends. Am J Gastroenterol.

[R16] Fung CP, Lin YT, Lin JC, Chen TL, Yeh KM, Chang FY, et al (2012). Klebsiella pneumoniae in gastrointestinal tract and pyogenic
liver abscess. Emerg Infect Dis.

[R17] Zhang S, Zhang X, Wu Q, Zheng X, Dong G, Fang R, Zhang Y, Cao J, Zhou T (2019). Clinical, microbiological, and molecular epidemiological
characteristics of Klebsiella pneumoniae-induced pyogenic liver abscess in
southeastern China. Antimicrobial Resistance & Infection Control.

[R18] Li W, Zhou Y, Yang Q, Liu J, Sun T, Zhang F, et al (2022). Trends in the antimicrobial resistance of Klebsiella pneumoniae
causing pyogenic liver abscess from 2011 to 2020: a multicenter study. Front Cell Infect Microbiol.

[R19] Siu LK, Yeh KM, Lin JC, Fung CP, Chang FY (2012). Klebsiella pneumoniae liver abscess: a new invasive syndrome. Lancet Infect Dis.

[R20] Tian LT, Yao K, Zhang XY, Zhang ZD, Liang YJ, Yin DL, et al (2014). Liver abscesses in adult patients with and without diabetes
mellitus: analysis of clinical characteristics, features of causative
pathogens, and prognosis. Diabetol Metab Syndr.

[R21] De Jesus, Marisa B., Marthie M ("Understanding). Ehlers, and Ricardo F. Dos Santos.

[R22] Qian Y, Wong C, Lai S, Chen H, He X, Sun L,et (2016). A retrospective study of pyogenic liver abscess focusing on
Klebsiella pneumoniae as a primary pathogen in China from 1994 to 2015. Scientific reports.

[R23] Wang JH, Liu YC, Lee SS, Yen MY, Chen YS, Wang JH, et al (1998). Primary liver abscess due to Klebsiella pneumoniae in Taiwan. Clin Infect Dis.

